# Experiences of Pregnant Women With a Positive HIV Status in Sub-Saharan Africa: Protocol for a Scoping Review

**DOI:** 10.2196/76971

**Published:** 2025-11-07

**Authors:** Esther Lydie Mbobnda Kapche, Sara Bibi Mitha, Firoza Haffejee

**Affiliations:** 1 Department of Basic Medical Sciences Durban University of Technology Durban South Africa; 2 Alan Pittendrigh Library Durban University of Technology Durban South Africa

**Keywords:** HIV and AIDS, pregnancy, antenatal, experiences, sub-Saharan Africa

## Abstract

**Background:**

The World Health Organization reports that HIV and AIDS remain a significant public health concern in sub-Saharan Africa, which hosts over 60% of the global population of people living with HIV. Among these, there were 1.2 million pregnant women with HIV in 2023 who likely experienced challenges related to the pregnancy itself, the fear of infecting their newborn babies, and issues related to HIV stigma and disclosure. However, their experiences have not been summarized to inform tailored interventions to assist them in their journey to delivery.

**Objective:**

This scoping review aims to synthesize the existing evidence related to the experiences of pregnant women with an HIV-positive status in sub-Saharan African countries.

**Methods:**

A review of the relevant literature will be guided using the Joanna Briggs Institute methodological framework for scoping reviews. Studies will be eligible for inclusion if they report on reactions to a new HIV diagnosis and the management of these reactions. All eligible studies from 2014 to 2025 in English and French will be included. The following databases will be used for the search: PubMed, MEDLINE, EBSCOhost (CINAHL with Full Text), Scopus, and Web of Science. All identified records will be collated and uploaded into EndNote X21 for management. Eligible studies will be screened by 2 independent reviewers, and disagreements will be solved through discussion. Data extracted via a validated data extraction form developed for this review will be analyzed using content analysis and presented in a narrative format. NVivo (version 12) will be used to facilitate the data analysis process. This protocol is aligned with the PRISMA-ScR (Preferred Reporting Items for Systematic Reviews and Meta-Analyses extension for Scoping Reviews) guidelines.

**Results:**

We will include studies on timely intervention with antiretroviral therapy, close medical monitoring, careful planning around delivery, and comprehensive support for both mother and child. The findings will be disseminated through medical education conferences and publications. The database search was completed in March 2025, and the results are expected to be published in December 2025.

**Conclusions:**

This review will provide a comprehensive narrative of the experiences of pregnant women with a positive HIV status in sub-Saharan Africa and how they react when they are first diagnosed with HIV, as well as how they manage the news. It is anticipated that there will be evidence of mixed reactions to and balanced management of a new HIV diagnosis among pregnant women.

**International Registered Report Identifier (IRRID):**

PRR1-10.2196/76971

## Introduction

The World Health Organization reports that HIV and AIDS remain a significant public health concern worldwide, with sub-Saharan Africa (SSA) disproportionately affected [1]. Worldwide, 39.9 million people were living with HIV in 2023, with 44% of all new HIV infections among women and girls and 62% of all new HIV infections in SSA. Moreover, approximately 1.3 million pregnancies occurred in 2023 among people with HIV worldwide, with high rates of HIV among pregnant women in SSA [2]. Efforts have been made to decrease the impact of HIV on people’s lives [3], including strides to reduce the HIV incidence in newborns. Prevention of mother-to-child transmission programs have been aligned with the Joint United Nations Programme on HIV/AIDS 90-90-90 targets launched in 2014 and updated in 2021 to 95-95-95, whereby 95% of HIV-positive people should know their HIV status, 95% of people diagnosed with HIV should receive sustained antiretroviral therapy (ART), and 95% of people on ART should achieve viral suppression by 2030 [4]. The success of ART has resulted in a diminution of the perinatal transmission of HIV [5].

However, there is still stigma and discrimination attached to HIV and AIDS. Moreover, the initial diagnosis of HIV and AIDS, similar to any other life-threatening disease, causes patients to experience several distressing psychological and emotional states of mind. These include denial, fear of the consequences, emotional shock, hopelessness, and guilt [6]. Previous studies have shown that people living with HIV are vulnerable to mental health problems, including depression [7-9]. Equally, pregnant women are twice as likely to develop depression [10]. This is because pregnancy is a period in which physiological, hormonal, social, and psychological changes occur [11]. These changes may cause more concern in women living with HIV. In addition to coping with the effects of pregnancy and dealing with the anxieties of childbirth [12,13], they have concerns related to HIV disclosure, stigma, and fear of infecting the unborn child [14]. This is exacerbated in unplanned pregnancies [15]. Therefore, understanding the lived experiences of newly diagnosed HIV among pregnant women is essential.

HIV infection in pregnancy is associated with increased maternal, fetal, and neonatal complications, which can lead to maternal and infant mortality [16,17]. Moreover, some pregnant women face unique challenges in accessing HIV care due to societal stigma and their personalized health needs [18], although most SSA countries have adopted compulsory HIV testing during antenatal visits [19]. Studies have reported that being diagnosed with HIV during pregnancy can negatively affect interpersonal relationships [20], leading to emotional turmoil due to the dual challenges of pregnancy and an HIV diagnosis [21], and amplify shock and disbelief in the case of a lack of symptoms despite the positive result [22]. Furthermore, partner disclosure becomes more difficult during this time [23] and can trigger negative reactions such as violence and separation [24].

Although efforts to prevent initial HIV infection in pregnant women are important in preventing perinatal transmission [25], equally important are efforts to ensure that pregnant women who receive the news of an HIV-positive status are able to manage it [21]. One could assume that the reactions to and the experiences of a diagnosis of HIV of any HIV-infected person are similar to those of HIV-infected pregnant women. However, unless this is explicitly investigated, knowledge of the extent to which the experiences can differ is limited. In addition, emotional and social challenges following an HIV diagnosis can lead to poor adherence to ART [26], whereas better handling of a new diagnosis of HIV could greatly buffer psychosocial and mental health and help facilitate health care seeking and retention [27]. This makes understanding pregnant women’s lived experiences in managing their HIV status essential to developing tailored interventions that can support their journey to delivery [5].

Despite studies describing the experiences of pregnant women with diagnoses of HIV, as of January 2025, a preliminary search of PROSPERO, the Cochrane Database of Systematic Reviews, *JBI Evidence Synthesis*, and the Open Science Framework indicates that there are no previous or ongoing scoping reviews that summarize pregnant women’s experiences related to HIV antenatal care (ANC) in SSA or their reactions to the news of a positive HIV status. One recent review conducted on HIV ANC focused on factors associated with HIV testing during ANC [19], and another was conducted on the childbirth experience of women living with HIV [14] but not during pregnancy or in SSA. Therefore, the proposed scoping review will explore the experiences of pregnant women with an HIV-positive status in SSA countries, with a focus on their reactions to the news as well as the disclosure of their status.

On the basis of the aforementioned objective, the following research questions have been developed:

1. What types of evidence have been published regarding the experiences of pregnant women with a positive HIV status?
2. What are pregnant women’s reactions to the news of their HIV-positive status?
3. What are the experiences of an HIV-positive status among pregnant women?

## Methods

We will conduct the proposed scoping review according to the Joanna Briggs Institute methodology for scoping reviews and adhere to the PRISMA-ScR (Preferred Reporting Items for Systematic Reviews and Meta-Analyses extension for Scoping Reviews) reporting guidelines [28].

### Registration

This review is registered with the Open Science Framework repository [29].

### Eligibility Criteria

#### Overview

Eligibility criteria for this scoping review will draw from the Joanna Briggs Institute mnemonic for the formulation of scoping review questions describing the population, concept, and context of the study [30]. For studies to be included, they should meet the following criteria: be available in full text in English or French; be related to HIV ANC; focus on reactions, experiences, and disclosure; and be published between January 2014 and February 2025. Studies will be excluded should they not be available in full text; focus on HIV among women in the postpartum period; not include reactions, experiences, or disclosure; be published outside the identified search period; or not be available in English or French. Textbox 1 summarizes the inclusion and exclusion criteria.

**Inclusion and exclusion criteria.**

**Inclusion criteria**

- Population: studies conducted on pregnant women living with HIV
- Concept: experiences of pregnant women, reactions to the news of an HIV diagnosis, and disclosure of HIV status
- Context: studies conducted in sub-Saharan Africa (SSA)
- Time frame: studies conducted from January 2014 to March 2025
- Language: studies published in English or French

**Exclusion criteria**

- Population: studies conducted on pregnant women with a negative HIV status, studies conducted on postpartum women, and studies conducted on any other women with an HIV-positive status
- Concept: factors associated with HIV antenatal care, prevalence and incidence of HIV antenatal care, and any other outcome different from the primary outcomes that refers to experiences of HIV-positive pregnant women
- Context: studies conducted outside of SSA
- Time frame: studies conducted before 2014
- Language: studies published in any other language apart from English or French

#### Population

For this scoping review, we will source all relevant peer-reviewed articles regarding pregnant women with a positive HIV status. We will exclude studies on pregnant women with a negative HIV status and on women with a positive HIV status in the postpartum period.

#### Concept

This review focuses on HIV antenatal experiences when the woman is either newly diagnosed with HIV or is a person living with HIV who is pregnant.

#### Context

The context of the study is SSA, which was chosen because the number of new HIV infections among adolescents and young women in the region remains exceptionally high [31].

#### Time Frame

Eligible studies conducted from January 2014 to March 2025 will be included because it was in 2014 that the Joint United Nations Programme on HIV/AIDS 90-90-90 target was launched.

### Types of Sources

We will seek all peer-reviewed literature related to the study objectives. We will consider experimental and quasi-experimental study designs, including before-and-after and interrupted time-series studies. In addition, we will include analytical observational studies, including prospective and retrospective cohort studies, case-control studies, and analytical cross-sectional studies, as well as descriptive observational study designs, including case series, individual case reports, and descriptive cross-sectional studies. Qualitative studies will also be included but not limited to any qualitative design. In addition, systematic reviews will also be considered for inclusion in this scoping review. However, gray literature (dissertations), conference abstracts, commentaries, study protocols, and book chapters will not be included. We will also conduct a hand search of all included articles’ reference lists to identify any additional relevant studies.

### Search Strategy

In this review, we will use a 3-step search strategy. First, we conducted an initial limited search in PubMed and examined the words in the title and abstract, index terms, and keywords of several papers to identify search terms (Multimedia Appendix 1). We will then devise the list of search terms and review these with the librarian at the Durban University of Technology to develop a full search strategy for PubMed. On the basis of those keywords, we will systematically search 4 electronic databases: PubMed and MEDLINE, EBSCOhost (CINAHL with Full Text), Scopus, and Web of Science. We will adapt the search strategy, including all identified keywords and index terms, for each included database or information source. We will review peer-reviewed journals for primary studies with a clear empirical base using qualitative, quantitative, and mixed methods addressing the research questions. In addition, we will search for articles through the “cited by” search. Once article review is completed, we will search the reference lists of all the included articles to identify additional articles.

We will also use the university’s interlibrary loan system to retrieve some of the full-text articles. We will thereafter send an email and then a reminder to the corresponding authors of articles whose full texts are not available. We will only send 2 reminders to authors as we want to complete the review in 3 months. If there is no response after a reasonable time, the articles will be excluded from the review. We will report the outcomes of contacting the authors in terms of how many reminders were sent, how many authors responded, and how many authors shared the requested full texts. The first search was conducted in March 2025, and an updated search will be conducted in June 2025 before the final synthesis.

To conduct the search, we will combine the following keywords: “HIV” AND “pregnancy” AND “sub-Saharan Africa.” We will use Boolean terms (AND, OR, and NOT) to separate the keywords. We will also use wildcards and truncations to accommodate variations in the different databases. The search strategy will be piloted by independently reviewing 10 articles to examine interrater reliability and resolve early issues before the full collection of articles. This is to check the appropriateness of selected electronic databases and keywords. We will modify the syntax where needed. We will use the services of an experienced information specialist to ensure that a robust review search strategy is followed. To compile all relevant evidence sources and identify and remove duplicate records, the EndNote X21 reference manager (Clarivate Analytics) will be used to import and manage eligible studies.

### Study and Source of Evidence Selection

Following the search, we will collate and upload all identified records into EndNote X21, and thereafter, we will use the Rayyan systematic review software (Qatar Computing Research Institute) to remove duplicates. Two authors will screen the titles and abstracts independently using Rayyan according to the eligibility criteria. We will then retrieve potentially relevant sources in full. Thereafter, 2 authors will assess the full texts of the identified reports independently against the inclusion criteria using Rayyan. The 2 screeners have received the following instructions: “Your role is to systematically screen the titles and abstracts (and potentially full texts) of studies identified in our literature search to determine their relevance and eligibility for inclusion in our review, based on the predefined inclusion and exclusion criteria. Please familiarise yourself with the review protocol, specifically the section detailing the inclusion and exclusion criteria. This document outlines the specific criteria that must be met for a study to be included in the review. Initially, screen titles and abstracts will be screened to determine if a study potentially meets the inclusion criteria. If there is any doubt, include the study for further screening. A study should be included if it meets all the inclusion criteria and none of the exclusion criteria. A study should be excluded if it fails to meet one or more of the inclusion criteria or if it meets one or more of the exclusion criteria. Document your screening decisions (include/exclude/maybe) and provide a brief reason for each decision.” We will record the reasons for the exclusion of sources of evidence that do not meet the inclusion criteria at the full-text stage. During screening, journal names, authors, and publication years will not be hidden from screeners because the Rayyan software does not blind such information. Any disagreements that arise between the reviewers at each stage of this process will be resolved through discussion until a consensus is reached. To minimize bias during the screening rounds, the 2 authors will independently screen all sources, and a coefficient of agreement will be calculated at each round. If the coefficient is <50%, the 2 screeners will discuss the eligibility criteria to obtain a better understanding.

### Quality Appraisal

Two authors will independently appraise the quality of all full-text peer-reviewed studies included in this review using the Mixed Methods Appraisal Tool version 2018 [32] by rating each item using “yes,” “no,” or “can’t tell.” We chose the Mixed Methods Appraisal Tool as it allows for the appraisal of qualitative, quantitative, and mixed methods studies using the same tool [32]. It has also undergone recent evaluations to inform its usefulness and content validity [33].

### Data Extraction

Two authors will independently extract data from papers included in the scoping review using a data extraction tool on Google Forms developed by the reviewers (Multimedia Appendix 2). The Microsoft Excel spreadsheet outputs will be shared among the authors for discussion and validation before data synthesis. They will extract specific details such as (1) metadata (authors, year of publication, and country), (2) study methods and sample size, (3) reactions, (4) experiences, (5) disclosure, and (6) any other significant findings. Before data extraction, the 2 screeners will receive the following instructions: “Extract data from each included study. Carefully extract all relevant data from the included studies, as outlined in the extraction form. Be thorough: Ensure that all required data points are extracted, even if they are not immediately obvious. Document any missing data: If a piece of data is missing, clearly document that in the extraction form. Record the source: Always indicate the source of the data (specific paragraph, table, figure).” They will also pilot-test the tool on 5 articles and then modify it if the extraction alignment is poor or if changes are needed. We will also revise it as necessary while extracting data from each included evidence source. The screeners will detail all modifications to the data extraction tool in the final scoping review. Finally, a Cohen κ coefficient will be calculated to measure the agreement between the 2 data extractors. A κ value of 1 will indicate perfect agreement, whereas a value of 0 will indicate an agreement no better than chance.

### Ethical Considerations

Ethics approval is not required at this stage, as we will synthesize published literature only.

### Data Analysis and Presentation

All the authors will be involved in the synthesis, and there will be no procedure to blind synthesists. One author will synthesize the extracted data, and the other 2 will validate the synthesis. The first author is a native French speaker and will read and understand all eligible studies published in French. We will adopt a descriptive approach, using thematic analysis for data summarization and reporting as suggested by Braun et al [34]. We will present the findings of the included studies related to the experiences and challenges encountered by pregnant women with a positive HIV status using a narrative approach. This approach was chosen because our research questions focused on understanding the experiences of pregnant women with an HIV-positive status in SSA, and narrative synthesis is well suited for exploring qualitative data and providing a rich, contextualized understanding. Given the heterogeneity of the potential studies, a narrative synthesis will allow us to explore the variations in findings across different study settings and populations. Although a meta-analysis could have been conducted for quantitative studies, the lack of comparable outcome measures across all studies made it unsuitable for our review. Therefore, a narrative synthesis was chosen to provide a more holistic interpretation of the findings. We will also report on any other emerging themes.

## Results

**Figure 1:**
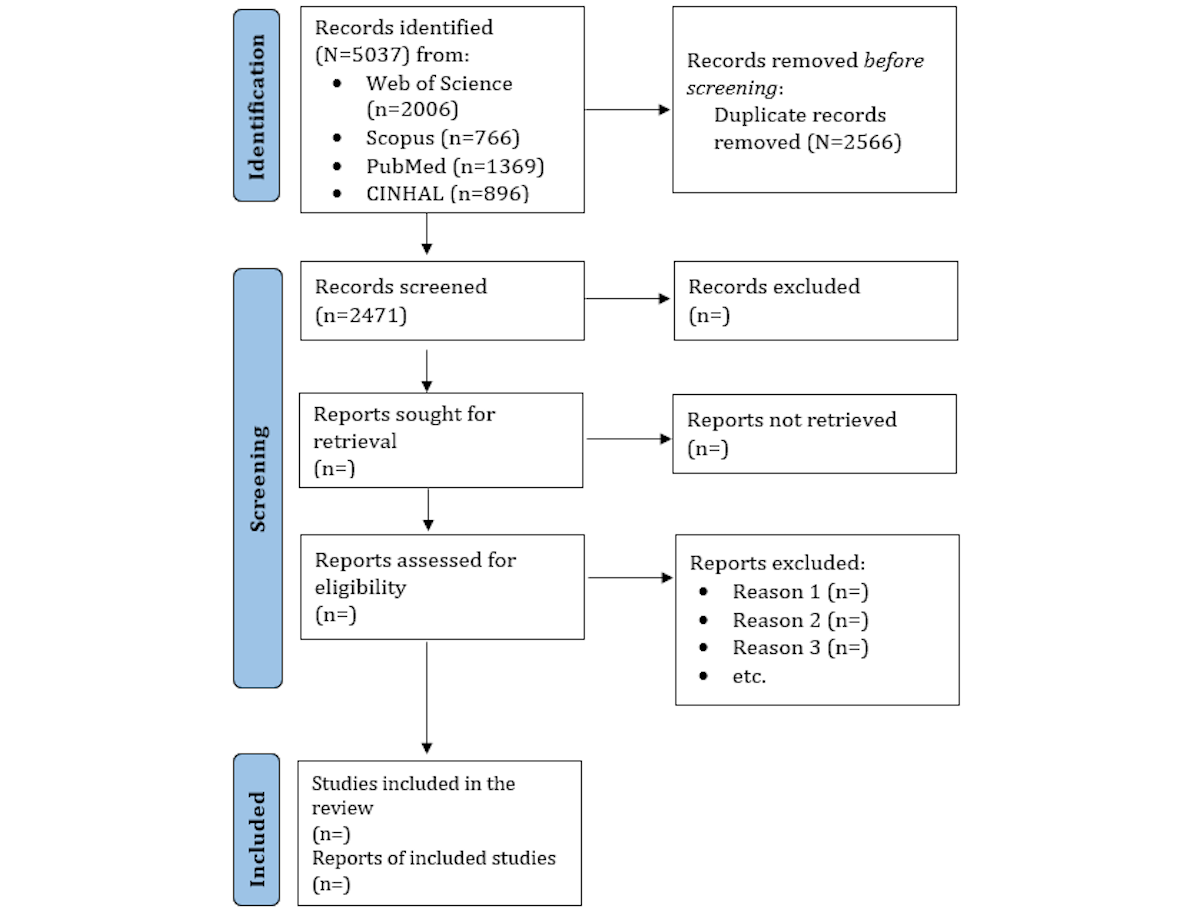
PRISMA (Preferred Reporting Items for Systematic Reviews and Meta-Analyses) flowchart. As of October 2025, the data search and screening of titles, abstracts, and full-texts have been completed.

Pregnant women’s reactions to and experiences with an HIV-positive status will be extracted from the included articles, and data will be analyzed to develop a comprehensive model for effective management of HIV during pregnancy that will include reports on timely intervention using ART, close medical monitoring, careful planning around delivery, and comprehensive support for both mother and child. As of May 2025, the database search and title and abstract screening have been completed. The results paper is expected to be published within the next 6 months. As of October 2025, the data search and screening of titles, abstracts, and full-texts have been completed. The results paper is expected to be published in December 2025. We will report the results of the search and the study inclusion process in a PRISMA-ScR flow diagram [35] (Figure 1).

## Discussion

### Expected Findings

This scoping review will be an overview of the current state of knowledge and research activities on the experiences of pregnant women with a positive HIV status in SSA, where HIV ANC has different characteristics. This review has the potential to contribute to achieving the United Nations’ goal of ending AIDS as a public health threat by 2030, as well as the Sustainable Development Goal (SDG) of reducing maternal and neonatal mortality related to AIDS by informing strategies to enhance culturally sensitive counseling and improve prevention of mother-to-child transmission outcomes. The results of this study will be, to the best of our knowledge, the first synthesis of the experiences of pregnant women with regard to HIV ANC. This review will identify challenges faced by pregnant women with a positive HIV status as HIV in pregnancy does not always mitigate the positive feelings toward childbirth and motherhood [14]. Moreover, some women discover their pregnancy and HIV-positive status simultaneously [21], placing them in a difficult position. The results of this review will also highlight strategies implemented by women to disclose their HIV-positive status to their families. Finally, the results of this review will inform a study that aims to address HIV-positive status in unplanned pregnancies in South Africa, which has one of the highest HIV prevalence rates worldwide, with approximately 7.5 million people living with HIV [36].

### Policy and Practical Implications

This scoping review aims to provide insights into the experiences of HIV-positive pregnant women in African countries. It will be instrumental in identifying key gaps and challenges in their pregnancy journey, as well as in HIV-related future research.

By linking the anticipated results to the SDGs, this review aligns with SDG 3, particularly targets 3.1 and 3.3, by addressing the intersecting challenges of maternal health and HIV. It also contributes to SDG 5.6 through its focus on reproductive health rights and gender-sensitive health care delivery. The findings will have both policy and practical implications for integrated HIV and maternal care services, stigma reduction, and improved retention in care for HIV-positive pregnant women. Health care providers are expected to benefit from the insights that this scoping review will provide regarding individualized needs for effective support of these women. This will improve HIV ANC. Furthermore, this review will emphasize the importance of community involvement in supporting and understanding HIV-positive journeys.

### Potential Challenges and Limitations

This review includes only studies published in English or French, which may exclude relevant research in other languages and limit the comprehensiveness of the findings. Furthermore, it focuses on studies published and indexed in selected databases, potentially overlooking unpublished research.

## Appendix

Multimedia Appendix 1 Pilot search.

Multimedia Appendix 2 Data extraction form.

## Abbreviations

ANC: antenatal care
ART: antiretroviral therapy
PRISMA-ScR: Preferred Reporting Items for Systematic Reviews and Meta-Analyses extension for Scoping Reviews
SDG: Sustainable Development Goal
SSA: sub-Saharan Africa

## Data Availability

All data generated and analyzed will be included in the published scoping review article and will be available on request.
