# Supplementary material for: Experiences of Pregnant Women With a Positive HIV Status in Sub-Saharan Africa: Protocol for a Scoping Review
Source: JMIR Res Protoc. 2025 Nov 7;14:e76971. doi: 10.2196/76971 (PMC12639334; doi:10.2196/76971)
Supplement: Multimedia Appendix 1 [file resprot_v14i1e76971_app1.docx]

**Appendix 1: Pilot search**

| **Search** | **Date of search** | **Search engine / Database** | **Number of publications retrieved** |
| --- | --- | --- | --- |
| Search: **((HIV) AND (pregnancy)) AND (sub Saharan Africa) AND (2014:2025[pdat])** Filters: **English, French, from 2014 - 2025** Sort by: **Most Recent**  (("hiv"[MeSH Terms] OR "hiv"[All Fields]) AND ("pregnancy"[MeSH Terms] OR "pregnancy"[All Fields] OR "pregnancies"[All Fields] OR "pregnancy s"[All Fields]) AND ("africa south of the sahara"[MeSH Terms] OR ("africa"[All Fields] AND "south"[All Fields] AND "sahara"[All Fields]) OR "africa south of the sahara"[All Fields] OR ("sub"[All Fields] AND "saharan"[All Fields] AND "africa"[All Fields]) OR "sub saharan africa"[All Fields]) AND 2014/01/01:2025/12/31[Date - Publication]) AND ((english[Filter] OR french[Filter]) AND (2014:2025[pdat]))  **Translations**  **HIV:** "hiv"[MeSH Terms] OR "hiv"[All Fields]  **pregnancy:** "pregnancy"[MeSH Terms] OR "pregnancy"[All Fields] OR "pregnancies"[All Fields] OR "pregnancy's"[All Fields]  **sub Saharan Africa:** "africa south of the sahara"[MeSH Terms] OR ("africa"[All Fields] AND "south"[All Fields] AND "sahara"[All Fields]) OR "africa south of the sahara"[All Fields] OR ("sub"[All Fields] AND "saharan"[All Fields] AND "africa"[All Fields]) OR "sub saharan africa"[All Fields] | 21 February 2025 | PubMed | 4287 |
