# Supplementary material for: Experiences of Pregnant Women With a Positive HIV Status in Sub-Saharan Africa: Protocol for a Scoping Review
Source: JMIR Res Protoc. 2025 Nov 7;14:e76971. doi: 10.2196/76971 (PMC12639334; doi:10.2196/76971)
Supplement: Multimedia Appendix 2 [file resprot_v14i1e76971_app2.docx]

**Appendix 2: Data extraction form**

| First Author’s name |  |
| --- | --- |
| Year of Publication |  |
| Country |  |
| Aim of the study |  |
| Study design |  |
| Sampling method |  |
| Sampling size |  |
| Reactions of pregnant women with a positive HIV status |  |
| Challenges faced by pregnant women with a positive HIV status with regards to the pregnancy |  |
| Challenges faced by pregnant women with a positive HIV status with regards to status disclosure |  |
| Strategies implemented by women to overcome their challenges (if any) |  |
| Other significant finding(s) |  |
